# Are life-extending treatments for terminal illnesses a special case? Exploring choices and societal viewpoints

**DOI:** 10.1016/j.socscimed.2017.12.019

**Published:** 2018-02

**Authors:** Neil McHugh, Job van Exel, Helen Mason, Jon Godwin, Marissa Collins, Cam Donaldson, Rachel Baker

**Affiliations:** a Yunus Centre for Social Business and Health, Glasgow Caledonian University, United Kingdom; b Institute of Health Policy & Management and Erasmus School of Economics, Erasmus University Rotterdam, Rotterdam, The Netherlands; c Institutes for Applied Health Research and Society & Social Justice Research, School of Health and Life Sciences, Glasgow Caledonian University, United Kingdom

**Keywords:** United Kingdom, End-of-life, Policy choices, Societal viewpoints, NICE, Life extension

## Abstract

Criteria used by the National Institute for Health and Care Excellence (NICE) to assess life-extending, end-of-life (EoL) treatments imply that health gains from such treatments are valued more than other health gains. Despite claims that the policy is supported by societal values, evidence from preference elicitation studies is mixed and in-depth research has shown there are different societal viewpoints. Few studies elicit preferences for policies directly or combine different approaches to understand preferences.

Survey questions were designed to investigate support for NICE EoL guidance at national and regional levels. These ‘Decision Rule’ and ‘Treatment Choice’ questions were administered to an online sample of 1496 UK respondents in May 2014. The same respondents answered questions designed to elicit their agreement with three viewpoints (previously identified and described) in relation to provision of EoL treatments for terminally ill patients. We report the findings of these choice questions and examine how they relate to each other and respondents' viewpoints.

The Decision Rule questions described three policies: DA – a standard ‘value for money’ test, applied to all health technologies; DB – giving special consideration to all treatments for terminal illnesses; and DC – giving special consideration to specific categories of treatments for terminal illnesses e.g. life extension (as in NICE EoL guidance) or those that improve quality-of-life (QoL). Three Treatment Choices were presented: TA – improving QoL for patients with a non-terminal illness; TB – extending life for EoL patients; and TC – improving QoL at the EoL.

DC received most support (45%) with most respondents giving special consideration to EoL only when treatments improved QoL. The most commonly preferred treatment choices were TA (51%) and TC (43%). Overall, this study challenges claims about public support for NICE's EoL guidance and the focus on life extension at EoL and substantiates existing evidence of plurality in societal values.

## Highlights

• In NICE technology appraisals life-extending, EoL treatments are a special case.
• Mixed findings have emerged from preference elicitation studies of societal values.
• We elicit EoL views and preferences for ‘Decision Rules’ and ‘Treatment Choices’.
• Respondents prioritise QoL improvements and findings challenge NICE's EoL guidance.

## 1 Introduction

In 2009 the National Institute of Health and Care Excellence (NICE) issued supplementary guidance for the appraisal of life-extending, end-of-life (EoL) treatments (NICE, 2009). This guidance permits such treatments to be recommended, even if they are not cost-effective according to usual standards, if certain criteria are met. These criteria are: 1) the treatment is for patients with short life expectancy normally less than 24 months, 2) the treatment would offer an extension to life of at least 3 months, and 3) the treatment is licensed for a small patient population (NICE, 2009). NICE, like other national Health Technology Assessment (HTA) organisations, has adopted an approach to economic evaluation based on cost utility analysis and applies a threshold cost per quality-adjusted life year (QALY) of £30,000 (NICE, 2013). For technologies that meet the EoL criteria a threshold of £50,000 per QALY has emerged over time (The Parliamentary Office of Science and Technology, 2015) implying that life-extending QALYs for patients with terminal illnesses are valued 1.7 times more than QALYs gained from all other types of treatment.

By raising the threshold for, or giving additional weight to, life-extending health gains at the EoL, the supplementary guidance suggests that these health benefits are of greater value (to society) than other types of health gains and that EoL might be considered a special case (Rawlins et al., 2010). However, empirical evidence of societal support for such a claim remains equivocal (Shah, 2017) and there is an opportunity cost to the EoL policy in terms of the health gains that would have arisen if spending had been allocated in other ways (Collins and Latimer, 2013). In this study, we examine societal preferences for provision of life-extending treatments for people with a terminal illness using two types of survey question, presenting respondents with choices between ‘Decision Rules’ (designed to reflect policies of the type that might be applied by national HTA organisations) and ‘Treatment Choices’ (of the kind that might be made by a regional health board with a fixed budget).

### 1.1 Background

Empirical studies that elicit societal values around EoL have deployed a variety of methods, including discrete choice methods (DCM) (Rowen et al., 2016, Shah et al., 2015a, Skedgel et al., 2015), person trade-off (PTO) (Pinto-Prades et al., 2014), budget allocation (Linley and Hughes, 2013) and willingness to pay (WTP) (Pennington et al., 2015, Pinto-Prades et al., 2014). The results of these studies have been remarkably mixed. In a recent literature review, Shah (2017) identifies 20 papers reporting empirical studies of societal values and EoL. Seven papers find a positive premium for EoL, nine negative and four report mixed findings (see Shah (2017) for a detailed summary of these papers). Of these studies only three examined preferences for different types of health benefit at the EoL; Pinto-Prades et al. (2014) and Shah et al. (2014) found a preference for quality-of-life improvements and Shah et al. (2015b) a preference for life extensions. The reason for such mixed findings is not clear (Shah, 2017) but it is difficult to explain wholly on the basis of study design, sampling or elicitation methods.

One possible explanation is that such mixed findings reflect substantial moral disagreement. This hypothesis is supported by the findings of the first phase of a two-phase study (funded by the Medical Research Council (MRC) Methodology Panel: project ID number G1002324) that used Q methodology to understand the nature of UK societal perspectives around the relative value of life extensions for people with a terminal illness (McHugh et al., 2015). Q methodology combines qualitative and quantitative methods to study ‘subjectivity’ – opinions, beliefs or values (Stephenson, 1953, Watts and Stenner, 2012). Data collection is via a card sort, and by-person factor analysis enables shared views around a given topic to be identified and then described; this methodology has previously been applied to the field of health (Baker et al., 2006, van Exel et al., 2015). Using this methodology we identified three viewpoints: i) ‘A population perspective – value for money, no special cases’, ii) ‘Life is precious – valuing life-extension and patient choice’, iii) ‘Valuing wider benefits and opportunity cost – the quality of life and death’. These viewpoints (described in detail in the original paper) highlight the plurality of views that exist in society around this topic and indicate that current NICE EoL policy may find little support. The viewpoints in this first phase of work are based around statements of opinion, principles and values relating to the provision of life-extending treatments at the EoL. As such, they are more abstract than most preference elicitation tasks, which tend to describe specific (albeit hypothetical) choices and trade-offs. A clear strength of preference elicitation studies is that opportunity cost is presented in terms of the choice foregone or through WTP (both imply budget constraints). However, preference elicitation scenarios are often attribute-based, can be brief or unrealistic and support for policy tends to be inferred by aggregating responses to these tasks. In the context of EoL, preference elicitation studies have resulted in such mixed findings that simple aggregation and measures of central tendency likely conceal heterogeneity. Combining preference elicitation with other approaches might shed light on both the mixed findings in the existing literature and whether respondents are consistent between their viewpoints and stated preferences.

On the rare occasions that different approaches have been combined to examine societal values and EoL, inconsistent results have been found. Rowen et al. (2014) presented attitudinal questions to respondents, following a series of choice-based questions designed as a Discrete Choice Experiment (DCE). Despite their DCE results indicating some support for an EoL premium, responses to attitudinal questions suggested limited support for life-extending treatments and patients at the EoL. Shah et al. (2015b) explored whether respondents agreed with the policy implications of their responses to stated preference tasks. Respondents were first asked to make choices between pairs of scenarios that were either abstract or ‘real-world’ resource allocation decisions (the latter included qualitative descriptions of patients' quality-of-life and information about the ages of patients instead of conceptual diagrams depicting information about patients, medical conditions and treatments as used in the former), and then were asked to state their agreement (or not) with the implied policy implication of their choice. Results suggested that some respondents struggled to align their views with the need to make specific trade-offs around prioritisation decisions and that disagreement with the policy implications of their choice could result from respondents differing interpretation of policies. These results imply that responses to specific choices and trade-offs may not align with more general beliefs or views around life-extending EoL treatments and that further exploration of this could help us understand the mixed, empirical EoL findings.

In this paper we examine responses to specific choices as well as agreement with more abstract viewpoints in relation to the provision of life-extending treatments for people with a terminal illness (‘terminal illness’ and ‘EoL’ are used interchangeably as the NICE supplementary guidance uses the term ‘EoL’ (NICE, 2009) and their definition implies ‘terminal illness’). Respondents were asked to make choices framed with respect to policies at a national level (‘Decision Rule’) and treatment provision at a regional level (‘Treatment Choice’). One scenario in each case is designed to mirror NICE EoL guidance. We elicit respondents' support for the societal viewpoints identified in our earlier Q methodological work (McHugh et al., 2015). We then examine how choices between Decision Rules and Treatment Choices correspond to each other and to the wider societal viewpoints.

## 2 Methods

### 2.1 Survey design

The survey was split into different versions, one of which incorporated the Decision Rule and Treatment Choice questions. In addition to these policy choice questions (described in more detail below) respondents were asked to give Likert scale responses (Viewpoint Questions) to indicate (dis)agreement with the three viewpoints identified in McHugh et al. (2015). The questionnaire concluded with socio-demographic questions. Appendix 1 shows the script used in the introductory animation and Appendices 2-4 detail the text of each question (accessed online at: http://www.gcu.ac.uk/endoflife/onlinesurvey/).

### 2.2 Decision rule design

The Decision Rule (D) question (see Appendix 2) was designed to represent the types of high-level rules applied to coverage decisions, at a national level, by bodies like NICE and the Scottish Medicines Consortium (SMC) in the UK. Specifically, respondents were asked to select how a health system should assess drugs for terminally ill patients that would not pass a standard ‘value for money’ (VFM) test (used as a lay term for cost effectiveness – see ‘Notes’ in Appendix 2 for definition). Respondents were then presented with a choice between three mutually exclusive policies: DA – a standard VFM test applied to all new health technologies (‘DA – standard VFM test’); DB – permitting ‘special consideration’ (i.e. provision of treatments even if the VFM is not passed) for all EoL treatments (‘DB – special consideration EoL’); and DC, permitting special consideration to specific categories of new treatments for terminal illnesses, such as those that extend life only or improve QoL only (‘DC – EoL … it depends’). DC is most like the NICE EoL supplementary guidance for those respondents selecting life extension as the specific reason for special consideration.

### 2.3 Treatment choice design

The Treatment Choice question (see Appendix 3) was designed to represent the types of decision that might be faced by Health Boards or Clinical Commissioning Groups, confronted with a fixed, additional budget. Respondents first selected their most-preferred treatment (A-C) then their second-best treatment and following this one reason (from a closed set of options) for their choice of most-preferred treatment. Next, respondents were presented with two PTO questions (pairing their most-preferred versus second-best treatments and their most-preferred versus least-preferred treatments). TA – improving quality-of-life for 100 patients with a non-terminal illness (TA – Non-EoL-QoL) episodically for the rest of their life; TB - extending life by three months for 100 EoL patients (TB – EoL-LE), and TC - improving symptoms for 100 patients in the last year of their life (TC – EoL-QoL). The size of the health gain from TA, TB and TC was implied rather than explicitly stated within the treatment descriptions.

The PTO questions required respondents to choose between providing treatment to one of two patient groups in the context of a fixed budget (only one patient group could be treated); the number of patients in each group was initially set as equal (100). Respondents were then asked to imagine that the cost of their preferred treatment changed, meaning fewer patients could be treated in their preferred group, while the other treatment could still treat 100 patients. The number of patients in their preferred treatment group was altered between low and high numbers of treated patients, until a point of indifference was reached (Nord, 1995).

### 2.4 Viewpoint questions design

The Viewpoint Questions were designed to measure respondents' agreement with one of three viewpoints, identified in earlier, in-depth research using Q methodology. The first viewpoint – *‘A population perspective – value for money, no special cases*’ – is a broadly utilitarian, system-level perspective. Importance is given to maximizing the health benefits, from a fixed health budget, to a population. Accordingly treatments that yield greatest health improvements in relation to cost should be prioritized and all patient groups should be considered equally deserving of treatment. The second viewpoint – *‘Life is precious – valuing life-extension and patient choice’* – is an individual patient perspective and is based on rights-based arguments and views about entitlement. Human life is considered precious and treatments should not be denied because of cost. Consequently no treatments are viewed as being a special case, rather the key criteria is that if a patient wants a treatment, including life-extending treatments at the EoL, they should have it because everyone contributes to the funding of the NHS. The third viewpoint – ‘*Valuing wider benefits and opportunity cost - the quality of life and death’* – is similar to the first as it recognizes the importance of achieving value for money from the health budget. However, this viewpoint also appreciates that there may be value for patients and their families from receiving treatment that goes beyond the measurable health benefits typically used in standard cost-benefit calculations. For more detail on these viewpoints see McHugh et al. (2015).

Salient and distinguishing statements from the original Q study were selected to characterise each of the viewpoints (see Appendix 4) following methods described in Baker et al., 2010a, Baker et al., 2014 and Mason et al. (2016). Six statements were identified to distinguish each of the three viewpoints, resulting in a set of 18 statements selected from the original 49 statements (McHugh et al., 2015). Crucially, these statements are used as ‘flags’ to signal allegiance with the whole viewpoint, they do not ‘sum up’ the viewpoint in its entirety.

Each of the 18 statements was presented to survey respondents, in random order, accompanied by a 7-point Likert scale labelled from “completely disagree” to “completely agree”. On completion, three scores were calculated for each respondent, indicating their level of agreement with each of the three viewpoints. Respondents were assigned to the viewpoint consistent with their highest score and to the category ‘mixed’ if their highest scores were equal on more than one viewpoint.

### 2.5 Data collection

The online survey was programmed and administered by YouGov (www.yougov.co.uk) and can be viewed and completed via the project website: http://www.gcu.ac.uk/endoflife/onlinesurvey/.

Prior to programming, survey questions were piloted in six focus groups with members of the public (n = 54), recruited via a market research company to ensure variation across socio-demographic characteristics (age, gender and income). In addition, prior to and after programming survey questions were piloted with a convenience sample of university colleagues to test the design, wording and comprehension of questions. Qualitative probing during the pilot led to a better understanding of how respondents interpreted the questions and question wording was amended accordingly. For example, in TA (Non-EoL-QoL) respondents asked if the duration of quality-of-life improvement was for the rest of the patients' life so the words “for the rest of their life” were added.

Respondents to the main survey were quota sampled from YouGov's UK online survey panel to represent the UK population on the basis of age, gender, socio-economic group (SEG) and ethnicity.

The survey was structured as follows: first, a short animated video (created specifically for this project) introduced and set the context for the survey (see http://www.gcu.ac.uk/endoflife/onlinesurvey/introductoryanimation/). The video describes, in simple terms, the issues of scarcity and opportunity cost within the NHS and the need to make decisions about the provision of treatments and services. It explains that many different things could be considered when making decisions about how best to allocate resources, such as severity of illness or quality-of-life, and that in this research the focus was on treatments that help terminally ill patients live longer (see Appendix 1).

Following the introduction, the 18 Viewpoint Questions were presented followed by the Decision Rule and then Treatment Choice questions. The survey finished with a number of socio-demographic questions.

### 2.6 Data analysis

As online surveys are susceptible to ‘clicking through’ and to respondents being distracted, those who completed the survey very quickly (less than 7 min and 30 s) or very slowly (longer than 2 h) were excluded from the analysis. Respondents who completed the survey in less than 7 min and 30 s, were considered to have reached completion too quickly to have fully read and understood the tasks. Similarly, those respondents who took more than 2 h to reach completion, might not have fully engaged with the survey and the time taken to complete may have inhibited their ability to recall the premise of the survey outlined in the introductory video. It is possible that, by imposing these rules, valid responses were excluded. However, these conservative cut-off times were based on the judgment of the research team informed by timed testing of the survey. Sensitivity analysis was conducted to examine the impact of imposing these exclusions on findings.

Summary statistics detailing frequencies for both the Decision Rule and Treatment Choice questions were calculated. Hypotheses about which choice of treatment would logically follow from respondents' decision rule are shown in Table 1.

**Table 1:** Hypotheses: Decision rule and treatment choice.

Decision Rule	Treatment Choice		
	TA (Non-EOL-QoL)	TB (EoL-LE)	TC (EoL-QoL)
DA (standard VFM test)	Yes	No	No
DB (special consideration EoL)	No	either potentially consistent	
DC (EoL … it depends)	No	either potentially consistent	

If respondents' choice of treatment reflects their decision rule those selecting Decision Rule DA would be more likely to choose Treatment Choice TA as this choice reflects a preference for maximizing health gains from a fixed budget. Respondents selecting DB favour all treatments for terminal illnesses so are likely to choose TB or TC. Similarly these two treatments – TB and TC – are also likely to be selected by those who prefer DC.

PTO ratios were calculated, reflecting respondents' strength of preference for treatment choices. While there is no single, correct approach for aggregating PTO ratios, there is consensus that one method – calculating the ‘mean of ratios’ – should be avoided. Following Baker et al., 2010b, Chilton et al., 2002, and Pinto-Prades et al. (2014), we calculated the ‘ratio of means’ and the ‘median of ratios’ (see Appendix 5 for details and illustrative calculations).

The relationship between respondents' decision rule and treatment choices and their viewpoints was also hypothesised (see Table 2). Similar predictions (as detailed in Table 1) were made about which choices of decision rule and treatment choices would logically follow from respondents' viewpoints.

**Table 2:**
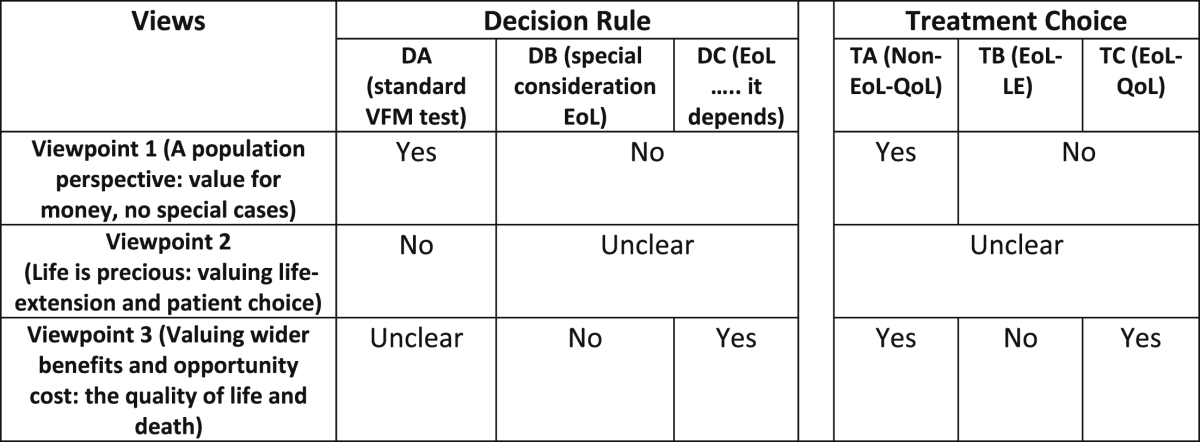
Hypotheses: Viewpoints and policy choices.

If choices reflect viewpoints then respondents associated with Viewpoint 1 (a broadly utilitarian account) would be more likely to choose Decision Rule DA and Treatment Choice TA because these choices would maximize health benefits. Respondents associated with Viewpoint 2 believe that patient choice is paramount and life is precious, and that cost should not drive decisions about treatment provision so no clear decision rule or treatment choice preference follow. We might predict, however, that those holding Viewpoint 2 would object to a strict value for money approach that overrules patient choice on the grounds that they reject consideration of cost. Respondents who agree most with Viewpoint 3 are likely to prefer DC, TA and TC as quality-of-life is reflected in these choices; DA could also be chosen because of value for money concerns but may be considered too narrow a decision rule for this account.

### 2.7 Research ethics

Ethical approval for this study was obtained from the School of Health and Life Sciences Ethics Committee, Glasgow Caledonian University (reference B11/04).

## 3 Results

### 3.1 Sample size, exclusion criteria and characteristics

Data were collected in May 2014. The full sample across all survey versions totaled 5496 respondents and was quota sampled from YouGov's online panel to be nationally representative with respect to age, gender, SEG and ethnicity. 1657 respondents were randomly allocated to the Policy Choice Version; after exclusion of respondents based on completion times the Policy Choice Version totaled 1496 (Table 3 details respondents socio-demographic characteristics in total and according to their policy choices). Sensitivity analysis showed that the use of exclusion rules made no difference to the percentages selecting options within the Decision Rule and Treatment Choice questions.

**Table 3:** Respondent characteristics: Total sample, Decision Rule and Treatment Choice.

Variables	Total Sample		Decision Rule							Treatment Choice						
			DA (standard VFM test)		DB (special consideration EoL)		DC (EoL … it depends)		Pˆ	TA (Non-EoL-QoL)		TB (EoL-LE)		TC (EoL-QoL)		Pˆ
	N	%	N	%	N	%	N	%		N	%	N	%	N	%	
**Gender**									0.1^a^*							<0.01^a^***
Male	**739**	**49.4**	282	52.8	145	49.5	312	46.6		386	50.6	54	63.5	299	46.1	
Female	**757**	**50.6**	252	47.2	148	50.5	357	53.4		377	49.4	31	36.5	349	53.9	
**Age**									0.03 a**							<0.01 a***
18-29	**247**	**16.5**	91	17	61	20.8	95	14.2		133	17.4	18	21.2	96	14.8	
30-49	**511**	**34.2**	165	30.9	105	35.8	241	36.0		256	33.6	42	49.4	213	32.9	
50-64	**380**	**25.4**	133	24.9	74	25.3	173	25.9		184	24.1	20	23.5	176	27.2	
65-74	**197**	**13.2**	78	14.6	29	9.9	90	13.5		97	12.7	4	4.7	96	14.8	
75+	**161**	**10.8**	67	12.5	24	8.2	70	10.5		93	12.2	1	1.2	67	10.3	
**Ethnicity**									0.02 a**							0.4 a
White	**1364**	**91.2**	497	93.1	254	86.7	613	91.6		701	91.9	74	87.1	589	90.9	
Non-white	**104**	**7**	30	5.6	31	10.6	43	6.4		50	6.6	9	10.6	45	6.9	
Prefer not to say	**28**	**1.9**	7	1.3	8	2.7	13	1.9		12	1.6	2	2.4	14	2.2	
**Country**									0.9^b^							0.3^b^
England	**1227**	**82**	439	82.2	239	81.6	549	82.1		627	82.2	73	85.9	527	81.3	
Wales	**80**	**5.3**	28	5.2	16	5.5	36	5.4		32	4.2	5	5.9	43	6.6	
Scotland	**165**	**11**	61	11.4	31	10.6	73	10.9		91	11.9	7	8.2	67	10.3	
Northern Ireland	**24**	**1.6**	6	1.1	7	2.4	11	1.6		13	1.7	0	0	11	1.7	
**SEG**^c^									0.02 a**							0.8 a
AB (SEG)	**461**	**31**	178	33.5	75	25.7	208	31.4		234	30.9	22	25.9	205	31.8	
C1 (SEG)	**451**	**30.3**	167	31.4	93	31.8	191	28.8		230	30.4	29	34.1	192	29.8	
C2 (SEG)	**234**	**15.7**	86	16.2	49	16.8	99	14.9		125	16.5	14	16.5	95	14.7	
DE (SEG)	**341**	**22.9**	101	19	75	25.7	165	24.9		168	22.2	20	23.5	153	23.7	
**Education Qualifications**									<0.01 a***							0.06 a*
Low qualifications	**350**	**23.4**	108	20.2	86	29.4	156	23.3		166	21.8	27	31.8	157	24.2	
Mid qualifications	**398**	**26.6**	132	24.7	77	26.3	189	28.3		198	26.0	20	23.5	180	27.8	
High qualifications	**712**	**47.6**	283	53.0	122	41.6	307	45.9		383	50.2	35	41.2	294	45.4	
Don't know	**9**	**0.6**	1	0.2	3	1.0	5	0.7		5	0.7	1	1.2	3	0.5	
Prefer not to say	**27**	**1.8**	10	1.9	5	1.7	12	1.8		11	1.4	2	2.4	14	2.2	
**Income**									<0.01 a***							0.8 a
Low income	**349**	**23.3**	106	19.9	82	28.0	161	24.1		180	23.6	19	22.4	150	23.1	
Middle income	**444**	**29.7**	163	30.5	80	27.3	201	30.0		237	31.1	25	29.4	182	28.1	
High income	**341**	**22.8**	148	27.7	57	19.5	136	20.3		178	23.3	15	17.6	148	22.8	
Don't know	**80**	**5.3**	26	4.9	18	6.1	36	5.4		36	4.7	4	4.7	40	6.2	
Prefer not to answer	**282**	**18.9**	91	17.0	56	19.1	135	20.2		132	17.3	22	25.9	128	19.8	
**Total**	**1496**		**534**	**35.7**	**293**	**19.6**	**669**	**44.7**		**763**	**51**	**85**	**5.7**	**648**	**43.3**	

^ˆ^ ‘Don't know’ & ‘Prefer not to say’ excluded from p-value calculation.

***1% significance level; **5% significance level; *10% significance level.

a Kruskal-Wallis tests.

b Chi-Square tests.

c n = 1487 because of missing data.

### 3.2 Relationship between policy choices and respondent characteristics

The relationship between respondents' socio-demographic characteristics and their policy choices (Decision Rule and Treatment Choice) is shown in Table 3. Respondents' gender, age, ethnicity, SEG, education and income were statistically significantly related to their choice of Decision Rule. Males were more likely to choose DA and females to choose DC; those selecting DB were younger than those selecting DA or DC and more-likely to be non-white; those with high educational qualifications, socio-economic status and income more often selected DA whereas respondents with low qualifications and income more often chose DB. Responses to Treatment Choice questions appeared to be related to gender, age, and education but in general, there were fewer statistically significant relationships. Males were more likely to choose TB and females to choose TC; those selecting TB were younger than those selecting TA or TC; and those with low educational qualifications chose TB whereas those with high qualifications selected TA.

### 3.3 Decision rule results

Table 4 shows the results from the Decision Rule question. DB was the least popular policy when respondents were asked ‘which one of the following policies do you agree with most?’, and overall there was a preference for giving special consideration to treatments for terminal illnesses in some (albeit not all) situations. Of the 669 respondents who selected DC, a large majority (72%) stated that treatments that improve quality-of-life for terminally ill patients should be given special consideration; only 10% stated special consideration should be given to treatments that extend life. When asked about the role cost should play in the provision of DB or DC, 56% of the 293 respondents who preferred DB thought this policy should be implemented regardless of cost, whereas 63% of the 669 respondents who preferred DC agreed there should be some limit to the amount the NHS pays to implement this policy.

**Table 4:** Decision rule results and reasons (n = 1496).

	DA (standard VFM test)	DB (special consideration EoL)	DC (EoL … it depends)	
Total selecting this policy	534 (36%)	293 (19%)	669 (45%)	

Valuing types of health gain	–	–	Improve QoL	485 (72%)
	–	–	Extend Life	64 (10%)
	–	–	Depending on. .^a^	120 (18%)

Regardless of cost	–	165 (56%)	246 (37%)	
Limit to cost	–	128 (44%)	423 (63%)	

a Special consideration depends on something else, either: patients having known about their terminal illness for only a short period of time; patients not having had their fair innings in terms of length of life; life extension only being valued if quality-of-life is not poor or another (entered) reason.

### 3.4 Treatment choice results

#### 3.4.1 Ranking results and reason for choice

Table 5 presents the ranking results from the Treatment Choice question. Just over half of respondents (51%) chose to provide TA from the additional available budget (quality-of-life-improving treatment for non-terminal illness (Non-EoL-QoL)). A substantial number of respondents (43%) preferred TC (quality-of-life-improving treatment for a terminal illness (EoL-QoL)); only 6% preferred TB (life-extending treatment for a terminal illness (EoL-LE)). Examination of second-choice treatments reveals that TB (EoL-LE) remains the least-preferred treatment, while more respondents prefer TC (EoL-QoL) (48%) to TA (Non-EoL-QoL) (35%). The majority of respondents placed TB in third place (78%), whereas TC was ranked third by the least number of respondents (9%).

**Table 5:** Treatment choice ranking results.

		Total (%)	Second choice (%)		
			TA (Non-EoL-QoL)	TB (EoL-LE)	TC (EoL-QoL)
First choice (%)	TA (Non-EoL-QoL)	**763 (51.0)**	–	93 (6.2)	670 (44.8)
	TB (EoL-LE)	**85 (5.6)**	38 (2.5)	–	47 (3.1)
	TC (EoL-QoL)	**648 (43.4)**	489 (32.7)	159 (10.6)	–
Total (%)		**1496 (100)**	**527 (35.2)**	**252 (16.8)**	**717 (47.9)**

Respondents also chose one reason for selecting their preferred choice of treatment. Respondents who most-preferred TA did so because it would provide a larger health benefit gain (34%), it would improve quality-of-life (26%) and the illness affects patients for the rest of their life (22%). Quality-of-life improvement was also the primary reason behind the preferred selection of TC (77%). Those preferring TB did so mainly because it would extend life (50%).

### 3.5 PTO results

PTO questions paired respondents' most-preferred versus second-best treatment (1 vs. 2) and most-preferred versus least-preferred treatment (1 vs. 3); the totals in Table 5 detail the aggregated order in which each of the three treatments was ranked. Data from both PTO questions – 1 vs. 2 and 1 vs. 3 – were combined in order to aggregate responses for each pair of treatments – TA and TB, TA and TC and TB and TC. Counts, ratios and ‘extreme preferences’ (taken as the number of respondents who consider that fewer than 10 patients receiving one treatment is equivalent to 100 patients receiving the other) are shown in Table 6.

**Table 6:** Treatment Choice PTO results.

X vs. Y	TA (Non-EoL-QoL) vs. TB (EoL-LE)	TA (Non-EoL-QoL) vs. TC (EoL-QoL)	TC (EoL-QoL) vs. TB (EoL-LE)
Prefer X (%)	763 (90%)	763 (54%)	648 (88%)
Extreme preference: <10X = 100Y	450 (59%)	335 (44%)	388 (60%)
Prefer Y (%)	85 (10%)	648 (46%)	85 (12%)
Extreme preference: <10Y = 100X	37 (44%)	330 (51%)	35 (42%)
Mean prefer X	33	65	35
Mean prefer Y	95	70	94
Ratio of means X:Y (Y = 1)	0.34	0.93	0.37
Median of ratios (Y = 1)	0.08	0.98	0.08
**Total**	**848**	**1411**	**732**^a^

a PTO 1 v 3 data is missing from one individual.

While the initial ranking of treatments (see Table 5) indicates the ordering of treatments, examination of PTO data provides insight into the magnitude of preferences between pairs of treatments. For the pair TA versus TC both (the ‘ratio of means’ and the ‘median of ratios’) are close to one, suggesting that respondents value the two treatments similarly (0.93 and 0.98). Ratios indicate greater strength of preference for TA or TC when compared to TB.

In an attempt to ‘unpack’ the data a little, Table 6 also shows 'extreme preferences’. While a substantial proportion of respondents' make these extreme choices, the most pronounced differences are seen in the pairings when either TA or TC is set against TB (59% v 44% and 60% v 42%). In pair TA versus TC, despite ratios suggesting a slight preference for TA over TC there is a greater proportion of extreme preferences among respondents who prefer TC than those who prefer TA (51% vs. 44%). This is balanced against the fact that a greater number of respondents prefer TA.

The results of Treatment Choice, like the Decision Rule questions, indicate a preference for quality-of-life improving treatments for both non-terminal and terminal illnesses compared to life-extending treatments for the terminally ill.

### 3.6 Relationship between decision rule and treatment choices

Table 7 cross-tabulates Decision Rule and Treatment Choices. As hypothesised (see Table 1), those who selected DA more often chose TA (70%) and to a lesser extent TC (27%); TB was rarely chosen. Those who selected DB most commonly chose TC (57%), as predicted, but, unexpectedly, more DB respondents selected TA (33%) than chose TB (10%). This pattern of response was the same for DC respondents: most chose TC (50%) as expected but a large number selected TA (44%) over TB (6%). This latter result is not wholly surprising, though, given that the majority of respondents who chose DC stated special consideration depends on improvements in quality-of-life (see Table 4).

**Table 7:** Relationship: Decision rule and treatment choice.

		Treatment Choice			Total
		TA (Non-EoL-QoL)	TB (EoL-LE)	TC (EoL-QoL)	
Decision Rule	DA (standard VFM test)	70.0%	2.8%	27.2%	534
	DB (special consideration EoL)	33.1%	10.2%	56.7%	293
	DC (EoL … it depends)	43.6%	6.0%	50.4%	669
**Total**)					**1496**

### 3.7 Relationship between viewpoints and policies

The results in Table 8 show that 37% of respondents were matched with Viewpoint 1 (‘A population perspective – value for money, no special cases’). Just under half of the respondents (49%) were matched with Viewpoint 2 (‘Life is precious – valuing life-extension and patient choice’) and 9% matched with Viewpoint 3 (‘Valuing wider benefits and opportunity cost – the quality of life and death’).

**Table 8:** Respondent viewpoints and policy choices.

Variables	Total Sample	Decision Rule				Treatment Choice			
		DA (standard VFM test)	DB (special consideration EoL)	DC (EoL … it depends)	Pˆ	TA (Non-EoL-QoL)	TB (EoL-LE)	TC (EoL-QoL)	Pˆ
	N	N (%)	N (%)	N (%)		N (%)	N (%)	N (%)	
Viewpoint					<0.01***				<0.01***
V1: A population perspective: value for money, no special cases	**558**	311 (55.7)	47 (8.4)	200 (35.9)		385 (69.0)	13 (2.3)	160 (28.7)	
V2: Life is precious: valuing life-extension and patient choice	**736**	128 (17.4)	218 (29.6)	390 (53.0)		258 (35.0)	66 (9)	412 (56.0)	
V3: Valuing wider benefits and opportunity cost: the quality of life and death	**141**	74 (52.5)	16 (11.3)	51 (36.2)		88 (62.4)	3 (2.1)	50 (35.5)	
Mixed	**61**	21 (34.4)	12 (19.7)	28 (45.9)		32 (52.5)	3 (4.9)	26 (42.6)	
**Total**	**1496**	**534 (35.7)**	**293 (19.6)**	**669 (44.7)**		**763 (51.0)**	**85 (5.7)**	**648 (43.3)**	

^ˆ^p-values calculated using Chi-Square tests (the ‘mixed’ category was excluded from the calculation).

***1% significance level.

Table 8 shows a statistically significant pattern between respondents' viewpoints and their policy choices; this pattern broadly reflects the hypotheses outlined in Table 2. Respondents associated with Viewpoint 1 were more likely to choose Decision Rule DA (56%) than DC (36%) or DB (8%) and, also as predicted, favour Treatment Choice TA (69%). Predictions were more difficult for Viewpoint 2 for reasons already mentioned. However, as expected, DA, which proposes a strict value for money approach, was the least preferred decision rule for those associated with Viewpoint 2 (17%). Unexpectedly respondents who were associated with Viewpoint 3 were more likely to select DA (53%) then DC (36%); few chose DB (11%). As predicted Viewpoint 3 respondents were more likely to choose a quality-of-life improving treatment – TA (62.4%) or TC (36%) – than one that extends life TB (2%). Respondents associated with Viewpoint 1 and Viewpoint 3 had a similar pattern of response to Decision Rule and Treatment Choice questions which could be a result of the relatively high correlation (0.68) between these viewpoints (McHugh et al., 2015). Although this may also relate to the relatively small number of respondents identified as Viewpoint 3 (n = 141).

## 4 Discussion

This paper reports the findings of a national survey of the UK general population investigating societal preferences for provision of life-extending treatments for people with a terminal illness framed with respect to policies at a national level (Decision Rule) and treatment provision at a regional level (Treatment Choice). Results challenge NICE's current EoL guidance as there is very little support for prioritising life-extending treatments for terminal illnesses over and above other treatments. Substantial support is found for quality-of-life improving treatments at the EoL and for policies which account for the costs of new treatments.

While Decision Rule findings showed the majority of respondents supported giving special consideration to assessing treatments for terminal illnesses (taking DC and DB together), this finding was qualified. More support was given to DC, which suggests that special consideration should be given to terminal illnesses in health care priority setting only in certain situations; with a focus on treatments that improve quality-of-life. Faced with Treatment Choices, respondents prioritized quality-of-life over life extension with preference for TA and TC over TB which aligned with the strength of preference results for these pairings; PTO results also indicated a substantial proportion of respondents made limited trade-offs (extreme preferences). Examining agreement with the three societal viewpoints indicated that our sample disagreed regarding the role cost should play in decision-making. While 49% of respondents were assigned to Viewpoint 2, an account that suggests that costs should not play a role in decision making, 46% of respondents (Viewpoint 1 and 3) recognized the importance of achieving value for money.

Exploration of the relationship between different Decision Rules and Treatment Choices found encouraging results as we observe broad consistency between respondents' preferences elicited from those choices. An unexpected observation was the proportion of respondents who selected Decision Rules that gave EoL treatments special consideration and preferred the Non-EoL-QoL health maximizing treatment (TA) (33% of those selecting DB and 44% of those selecting DC). This could be explained by the health gains of TA arising over patients' lifetimes and so respondents might reasonably have interpreted these as far exceeding the health gains likely to arise from 100 patients receiving either TB or TC. While this could indicate a disconnect between different types of preference, it could also be the case that preference for EoL is outweighed if the health gain from the alternative treatment is substantial. Unfortunately the online nature of our survey meant these issues could not be explored qualitatively.

Examining the pattern of response between respondents' viewpoints and their choices indicates that while in the majority of cases there is a pattern in line with expectations; it is not always the case. However, a priori hypotheses are not straightforward because viewpoints are wider and take in other issues, and choices were designed to examine support for NICE EoL policy rather than to mirror viewpoints exactly. Despite this limitation, interesting findings emerged. Results suggest that when respondents make choices they are more attuned to the limits of the NHS budget than when responding in more general terms when opportunity cost is not always explicit (Viewpoint Questions). More research is needed to examine the nature of consistency between principles, policies and choices, if policy is to be designed in areas of societal disagreement. As well as future work qualitatively exploring inconsistencies, the separation of preferences into different ‘levels’ – principles, policies and choices – of specificity and abstraction would enable examination of why and how respondents (dis)agree and whether there is potential for agreement in more-specific cases in the face of disagreement at the level of theory or principle (Sunstein, 1995).

The empirical literature eliciting societal values with respect to provision of EoL treatments has, to date, produced very mixed results with a similar number of papers reporting an EoL premium or no evidence of such a premium (Shah, 2017). Our survey methods, grounded in a previous, in-depth study of the nature of perspectives (McHugh et al., 2015), suggest a substantial proportion of the population (roughly a third of our sample) has broadly utilitarian motivations, preferring policies promoting cost effectiveness and maximizing health gains. In contrast to current NICE policy, which favours *life-extending* treatments at the EoL, our results also suggest that policies and treatments that prioritise quality-of-life are more important to the general population than those that prioritise length of life. On the basis of these findings, and the findings of Pinto-Prades et al. (2014) and Shah et al. (2014), life extension appears to be less valued by the public than quality-of-life at the EoL. This is an important observation given that media reports and other policy initiatives (e.g. Cancer Drugs Fund) might suggest that society values life-extending treatments above other treatments and services competing for funds. If additional societal benefits are not generated from prioritising funding for life-extending treatments at the EoL, then cost-effective treatments for non-EoL patients may be displaced by policies that prioritise less efficient treatments.

### 4.1 Limitations

There are a number of limitations to the study design and details of the survey that should be acknowledged. Firstly, respondents were quota-sampled to represent the general population with respect to standard socio-demographic variables, but they were members of YouGov's online survey panel, which introduces self-selection bias. Furthermore, a trade-off of undertaking large scale survey work is that we were unable to collect qualitative data alongside the main survey. As such, we could not explore qualitatively: why, in some cases, the relationships between Decision Rule and Treatment Choice responses, or between policy choices and viewpoints, were not as expected; whether and to what extent respondents viewed TA (Non-EoL-QoL) as the health maximizing treatment; nor respondents' rationales for their treatment choices to move beyond the circularity of re-stating details of the scenarios chosen.

Secondly, question ordering may have affected responses. We took the view that there was need for a consistent ordering as Viewpoint Questions introduce different issues related to the topic, and Treatment Choice selection was considered to flow more naturally from the choice of a high-level Decision Rule, but we cannot rule out the ordering effects that may have followed from this design.

Thirdly, the Decision Rule question could have introduced a ‘status quo’ framing effect by *stating* that the NHS *currently* applies a ‘value for money’ test before agreeing to provide new medicines. Thus respondents might have seen this as a choice between the status quo (DA) and something new – DB or DC – although the status quo is in reality closer to DC (along with a preference for life extension). Respondents could have been unwilling to choose a policy that contradicts the current agenda. However, our results show that DC (with a quality-of-life preference) was the most popular choice.

Fourthly, as shown in Table 2, no viewpoints clearly correspond to a preference for DB or TB because none of the three viewpoints from our original study would clearly predict those decision rule or treatment choices (McHugh et al., 2015). DB was included to examine if respondents valued *all* new treatments for terminal illnesses and TB represents NICE's current EoL policy.

Fifthly, in the introduction to the Decision Rule question the terms ‘drugs’ and ‘treatments’ could be construed as being interchangeable; this was not our intention and a clearer differentiation should have been made between the two as per NICE technology appraisal guidance (NICE, 2017). Whilst this could have led to different interpretations amongst respondents, we were given no indication of this during the piloting phase and the decision rule and treatment choice scenarios all refer to ‘treatments’. The focus on treatments is similar to the approach used in the EoL preference elicitation literature summarized in the ‘Background’ section. However, whether ‘treatment’ refers to drugs or something else is generally unstated in these papers; exploring preferences for different types of treatment at the EoL could be an interesting source of future research.

Lastly, respondents were assigned to a viewpoint based on their highest aggregate score on associated statements. While this gives an indication of what viewpoint respondents are most like it does not account for respondents being closely associated with multiple viewpoints. Given that quality-of-life receives substantial support from the policy choice questions, this could help to explain the unexpectedly small proportion of respondents (9%) assigned to Viewpoint 3.

## 5 Conclusion

This study challenges NICE's current EoL guidance and contributes, through use of innovative methods, to the growing body of empirical evidence around this topic. Elicited preferences indicate that for policy to better reflect societal values consideration needs to be given to quality-of-life improving treatments at the EoL and to the cost of new treatments. Our findings also caution against simplistic approaches to summarizing societal values using measures of central tendency. The methods used reveal plural societal views and that different relationships can exist between societal viewpoints and preferences expressed at different levels of specification. Future research should combine qualitative and quantitative methods to better understand the nature and distribution of societal values across different levels of specificity and abstraction. There would appear to be great potential in developing empirical studies of societal values that combine health economic and ethics based approaches, examining the relationship between principles/arguments and choices/preferences.

## Conflicts of interest

None.
