# Supplementary material for: Are life-extending treatments for terminal illnesses a special case? Exploring choices and societal viewpoints
Source: Soc Sci Med. 2018 Feb;198:61–9. doi: 10.1016/j.socscimed.2017.12.019 (PMC5884317; doi:10.1016/j.socscimed.2017.12.019)
Supplement: Supplementary material [file mmc1.docx]

**Appendix 1 Script for Introductory Animation**

***see** [**http://www.gcu.ac.uk/endoflife/onlinesurvey/introductoryanimation/**](http://www.gcu.ac.uk/endoflife/onlinesurvey/introductoryanimation/*) **for introductory animation***

1. The National Health Service is funded directly by the public.

2. The NHS spends its budget on many things, including doctors, nurses, beds, new drugs and treatments.

3. Although the health service budget is very big, it is still a fixed amount. There is never enough money to do everything we want.

4. Of course, the NHS budget could grow in the future.

5. But this research is about the money the health service has now, and the best way to spend it.

6. Because the budget is fixed, difficult decisions have to be made about how to spend NHS money.

7. When the NHS provides a service, the public benefits. But the public will not benefit if that service is not funded.

8. Because of this, and because the public pays for the NHS through its taxes, it is important that decisions on how to spend NHS money take into account the views of the public.

9. For example, thinking generally about all NHS patients, should we concentrate our funding on the treatment of people who are most severely ill? Or perhaps we should focus our spending on treatments that give people a better quality of life? Or should we prioritise the funding of treatments that help people to live longer?

10. In this research project, our focus is on NHS treatments that help terminally ill patients live longer.

11. These treatments will not cure the person’s illness, but will extend their life, usually by weeks or by months.

12. Whatever money is spent on these treatments is not then available to spend elsewhere in the NHS.

13. Because of this, the cost of treatments for terminally ill people, and how much good they do, has to be considered in relation to all other NHS spending.

14. These are difficult decisions to make, and there are no right or wrong answers.

15. As a member of the public, we need to know your views on this important topic.

**Appendix 2 Decision Rule Question**

***italics highlight programming notes***

As there is a limit to the health budget, the NHS applies a ‘value for money test’ before agreeing to provide new medicines. This test looks at the extra costs and the health benefits of new medicines compared with the best treatments already provided by the NHS, for that condition.

The value for money test makes sure the NHS does not spend money on high cost treatments, which provide only limited health benefits, but funds those treatments that deliver better value for money.

There are some drugs, for terminally ill patients, which do not pass this value for money test, because they do not cure patients. These medicines might extend life or improve quality of life for patients for a short period of time, but often at quite high cost.

The NHS is considering different ways of assessing drugs like these. We want to know what you think of a number of different policies.

It is important to keep in mind that if money is spent on these medicines for people with terminal illnesses, it is not available to spend on other treatments in the NHS.

**Q1**. Which one of the following policies do you **agree with most**? Please click the box below the policy you agree with.

| **POLICY A**  ***All*** new treatments, including those for terminal illnesses ***must*** pass the same value for money test. Only those treatments that pass the test are provided from the NHS budget. | **POLICY B**  ***All*** new treatments for terminal illnesses should be given special consideration (a different value for money test should be applied for new treatments for terminal illnesses). | **POLICY C**  It depends.. ***some*** new treatments for terminal illnesses should be given special consideration (a different value for money test should be applied for new treatments for terminal illnesses). |
| --- | --- | --- |
| 🞏 | 🞏 | 🞏 |

*If respondent ticked box:*

***A*** *– finish.*

***B*** *– go to Q2.*

***C*** *– go to Q4.*

**Q2.** In response to Q1 you selected Policy B.

You said that ***all*** new treatments for terminal illnesses should be given special consideration (a different value for money test should be applied for new treatments for terminal illnesses).

Which of the following do you **agree with most** (please tick only one box)?

- all new treatments for terminal illnesses should be given special consideration, regardless of their cost
- all new treatments for terminal illnesses should be given special consideration, but there should be a limit to the amount the NHS will pay

**Q3.** In response to Q1 you selected Policy B.

You said that ***all*** new treatments for terminal illnesses should be given special consideration (a different value for money test should be applied for new treatments for terminal illnesses).

Health benefits can either extend life or improve the quality of life. Which of the following do you **agree with most** (please tick only one box)?

🞏 Treatments for terminal illnesses that increase the length of a patient’s life are more important than those that improve a patient’s quality of life.

🞏 Treatments for terminal illnesses that improve a patient’s quality of life are more important than those that increase the length of a patient’s life.

🞏 Treatments for terminal illnesses that increase the length of a patient’s life or that improve quality of life are both equally important

🞏 Life extending treatments are more important, but they should only be provided if a patient’s quality of life is good

**Q4.** In response to Q1 you selected Policy C.

You said it depends.. ***some*** new treatments for terminal illnesses should be given special consideration (a different value for money test should be applied for new treatments for terminal illnesses).

Which policy do you **agree with most**? You can tick only one box.

| **POLICY C1**  New treatments that extend life for terminally ill patients should be given special consideration. | **POLICY C2**  New treatments that improve quality of life for terminally ill patients should be given special consideration. | **POLICY C3**  New treatments for terminally ill patients should only be given special consideration ***depending on*** (something else)  *Please go to Q5* |
| --- | --- | --- |
| 🞏 | 🞏 | 🞏 |

*Only respondents who selected Policy C3 answer Q5. Respondents selecting Policy C1 or C2 move on to Q6.*

**Q5.** In response to Q4 you selected Policy C3. You said treatments for terminally ill patients should only be given special consideration ***depending on*** *(something else)****. . . .***

Which policy do you **agree with most**? You can tick only one box.

| **POLICY C3a**  Treatments that extend life for terminally ill patients should be given special consideration (but not if their quality of life will be poor). | **POLICY C3b**  Treatments for patients who have only known that they were terminally ill for a short time should be given special consideration. | **POLICY C3c**  Treatments for terminally ill patients who have not had their fair innings - in terms of the length of their life should be given special consideration. | **POLICY C3d**  Treatments for terminally ill patients should be given special consideration ***if*** . . . . .  *Please type in your own answer in the space below* |
| --- | --- | --- | --- |
| 🞏 | 🞏 | 🞏 | 🞏 |

You selected Policy C3d in Q5, please insert your reason for this selection below.

**“**Treatments for terminally ill patients should be given special consideration if . . . . .

**Q6.** In response to Q4/5 you selected Policy ‘*insert corresponding policy letter from either Q4 or Q5’*.

You said (*insert policy letter and description from Q4/5)*

Which of the following do you **agree with most**, please tick only one box.

- Policy ‘*insert corresponding policy letter from either Q4 or Q5’* should be given special consideration, regardless of their cost
- Policy ‘*insert corresponding policy letter from either Q4 or Q5*’ should be given special consideration, but there should be a limit to the amount the NHS will pay.

**Notes**

In the programmed question respondents could mouse-over the following terms for definitions:

**Value for money test**: “The value for money test compares the extra costs and benefits of new medicines compared to existing treatments and makes sure the NHS does not spend money on high cost treatments that provide only limited health benefits.”

**Pass the test:** “There are some drugs for terminally ill patients near the end of their life which do not pass this value for money test, because they do not cure patients and so health benefits are quite limited, often at high cost.”

**NHS budget:** “If NHS money is spent on these medicines for people with terminal illnesses, it is not available to spend on other treatments in the NHS.”

**Special consideration:** “Special consideration means that treatments for people with terminal illnesses are provided even if they do not pass the value for money test that all other treatments must pass. There may still be limits, but those limits would be higher than for other treatments”.

**Terminal illness:** “Terminal illnesses cannot be cured and will lead to patients’ death, usually within a year.”

**Appendix 3 Treatment Choice Question**

***italics highlight programming notes***

The NHS has a fixed, additional budget available to spend on treatments for people in your area and is considering which of **three new treatments** to provide from this money. **Only one treatment can be provided**.

Below are descriptions of the three treatments. Which treatment should be provided?

**TREATMENT A**

Patients are currently suffering from a non-life threatening illness that causes them discomfort and fatigue. The illness also reduces their mobility and ability to undertake their usual activities. This occurs a few times throughout every year for the rest of their life. Each episode lasts for up to 2 weeks.

A new treatment is available that will reduce their symptoms and make patients feel better, improving their quality of life for the rest of their life.

Funding will mean that 100 patients can be treated in the next year.

**TREATMENT B**

Patients are currently suffering from a terminal illness that causes them discomfort and fatigue. The illness also reduces their mobility and ability to undertake their usual activities.

A new treatment is available for terminally ill patients in the last year of their life. The treatment will extend patients’ lives by three months. It will not improve their quality of life.

Funding will mean that 100 patients can be treated in the next year.

**TREATMENT C**

Patients are currently suffering from a terminal illness that causes them discomfort and fatigue. The illness also reduces their mobility and ability to undertake their usual activities.

A new treatment is available for terminally ill patients in the last year of their life. The treatment will reduce their symptoms and make patients feel better, improving their quality of life. It will not extend their life.

Funding will mean that 100 patients can be treated in the next year.

Q1. Which treatment should be provided? Click on the ‘Treatment’ you think should be provided.

Now from the remaining two treatments click on the ‘Treatment’ you think should be provided.

*Show the treatments on the screen in the order the respondent preferred them*

If only one treatment could be funded, you said that Treatment (*insert most preferred*) should be provided.

Treatment (*insert second most preferred*) was your next most preferred after treatment (*insert most preferred*).

Is that correct?

Yes/ No – (*if No go back and choose again*)

Q2.

*Show description of their ’preferred treatment from Q1’*

What was the most important reason for selecting [Treatment *insert treatment letter A-C]* for funding? Tick only one box.

1. End of life treatments are very important 🞏
2. The treatment will extend life 🞏
3. The treatment will improve quality of life 🞏
4. The illness affects patients for the rest of their life 🞏
5. I preferred supporting the non-life threatening illness 🞏
6. The patients with terminal illnesses will die anyway 🞏
7. This treatment will provide a larger health benefit gain 🞏
8. Other , please specify 🞏

_____*space to specify ‘other’*_________________

Q3a. Your ‘preferred treatment’ from Q1 was Treatment ‘*insert letter of preferred treatment’* and your **‘**second-best treatment’ was Treatment ‘*insert letter of second-best treatment’*. Only one treatment can be funded.

Suppose the cost of your ‘preferred treatment’, Treatment ‘*insert letter of preferred treatment’*, has now changed, and fewer patients can be treated as a result.

While your ‘second-best treatment’, Treatment ‘*insert letter of second-best treatment’*, will still treat 100 patients, (if funded), the number of patients that your ‘preferred treatment’, Treatment ‘*insert letter of preferred treatment’*, is now less.

When 100 patients could be funded by each treatment, you chose to fund Treatment ‘*insert letter of preferred treatment.*

Your choice is shown by the black circle below.

| Choice *[check box]* | *Insert Short Description of ‘preferred treatment’* | *Insert Short Description of ‘second – best treatment’* | Choice  *[check box]* |
| --- | --- | --- | --- |
|  | 100 patients | 100 patients |  |

What if only 10 patients could be treated with Treatment ‘*insert letter of preferred treatment’?* Would you prefer to fund 10 patients with Treatment ‘*insert letter of preferred treatment’* or would you prefer to fund 100 patients with Treatment ‘insert *letter of second-best treatment’?* Please indicate your preference by clicking on the [check box] below.

| Choice  *[check box]* | *Insert Short Description of ‘preferred treatment’* | *Insert Short Description of ‘second – best treatment’* | Choice  *[check box]* |
| --- | --- | --- | --- |
|  | 10 patients | 100 patients |  |

*[In this example, respondent chose ‘second-best treatment’ – shown as the black circle. Following response to the above questions, a full table appears with the second row and last row completed based on the above two responses, all other numbers in the table should appear but be greyed out.]*

What if we could only fund 90 patients with Treatment ‘*insert letter of preferred treatment’?* Would you prefer to fund 90 patients with Treatment ‘*insert letter of preferred treatment’* or would prefer to fund 100 patients with Treatment ‘insert *letter of second-best treatment’?* Please indicate your preference by clicking on the corresponding [check box] below.

| Choice  *[check box]* | *Insert Short Description of ‘preferred treatment’* | *Insert Short Description of ‘second – best treatment’* | Choice  *[check box]* |
| --- | --- | --- | --- |
|  | 100 patients | 100 patients |  |
|  | *90* | *100* |  |
|  | *80* | *100* |  |
|  | *70* | *100* |  |
|  | *60* | *100* |  |
|  | *50* | *100* |  |
|  | *40* | *100* |  |
|  | *30* | *100* |  |
|  | *20* | *100* |  |
|  | *10* | *100* |  |

*[This question repeats replacing the number for the ‘preferred treatment’ option with numbers from the second column alternating between, 20, 80, 30, 70, 40, 60, 50]*

From the table above you would prefer Treatment (*insert letter of second-best treatment*) when (*insert number of patients when the black circle moves to the right hand column – 60 in the above example*) patients would be funded by Treatment *(insert* *letter of second-best treatment)*.

Now, suppose there were *(insert x)* patients that could be funded by *(insert preferred treatment)* and 100 patients who could be funded by *(insert second best treatment)* which would you choose to fund?

*(note – x is the midpoint between: the lowest choice on the left hand column and the highest choice in the right hand column)*

| Choice  *[check box]* | *Insert Short Description of ‘preferred treatment’* | *Insert Short Description of ‘second – best treatment’* | Choice  *[check box]* |
| --- | --- | --- | --- |
|  | [x] patients | 100 patients |  |

Q3b *[only for respondents whose choice is their ‘preferred treatment’ option at 10]*

You said that you would prefer that 10 patients are funded by Treatment ‘*insert letter of preferred treatment’* compared to funding 100 patients with Treatment ‘insert *letter of second-best treatment’.*

Now, suppose there was only 1 patient that could be funded by *(insert preferred treatment)* and 100 patients who could be funded by *(insert second best treatment)* which would you choose to fund?

| Choice  *[check box]* | *Insert Short Description of ‘preferred treatment’* | *Insert Short Description of ‘second – best treatment’* | Choice  *[check box]* |
| --- | --- | --- | --- |
|  | 1 patient | 100 patients |  |

*[if choice = preferred treatment then end; if second preferred then present again but with 5 patients on left and 100 on right]*

*REPEAT FOR COMPARISON BETWEEN MOST PREFERRED AND LEAST PREFERRED.*

# Appendix 4 Viewpoint Questions: statements selected for survey (by viewpoint)

| Statement Number (from initial Q study) | **Viewpoint 1** | |
| --- | --- | --- |
| 3 | Treatments should be directed towards people who have a greater chance of survival. | |
| 5. | At the end of their life, patients should be cared for at home with a better quality of life rather than have aggressive and expensive treatments that will only extend life for a short period of time. | |
| 26. | It is wrong to raise hopes and expectations by making a special case for treatments that will only extend life by a short time. | |
| 38. | The health system should be about getting the greatest benefit overall for the population. | |
| 2. | We should support an individual patient's choice for treatments that give short life extensions. | |
| 13. | I would place more value on end-of-life treatments than many medical treatments for non-terminal conditions. | |
|  | |  |
|  | **Viewpoint 2** | |
| 17. | If a life-extending treatment for terminally ill patients is expensive, but the only treatment available, it should still be provided. | |
| 20. | We all have the right to life. | |
| 27. | To extend life in a way that is beneficial to the patient is morally the right thing to do. | |
| 37. | All human life is precious. | |
| 1. | It is not worthwhile devoting more and more NHS money to someone who is going to die soon anyway. | |
| 33. | End-of-life drugs are not a cure, they are life-prolonging. There is no point in delaying the inevitable for a short time. | |
|  | |  |
|  | **Viewpoint 3** | |
| 25. | We should spend proportionately more on patients when we feel those patients have not had their fair innings - in terms of the length of their life or the quality of that life. | |
| 31. | Treatments that are very costly in relation to their health benefits should be withheld. | |
| 34. | Patients at the end of life will grasp any slightest hope but that is not a good reason for the NHS to provide costly treatments that may extend life by a short time. | |
| 41. | I wouldn’t want my life to be extended just for the sake of it - just keeping breathing is not life. | |
| 23. | A year of life is of equal value for everyone. | |
| 24. | You can't put a price on life. | |

***Grey shaded statements are negatively associated with the viewpoint.***

**Appendix 5 An example of the two methods used to aggregate PTO responses**

Calculation of the ‘ratio of means’ involves assigning a value of 1 to the most-favoured treatment in each individual choice, with the less-favoured treatment receiving a value equal to the number of patients in the most-favoured group divided by the number of patients in the less-favoured group. Means across all respondents for each treatment (X and Y) are then calculated and then ratio of means determined. ‘Median of ratios’ comprises calculating ratios, X/Y (alternatively Y/X could be utilised), for each individual respondent and then taking the median of ratios across all respondents. These calculations are adapted from Pinto-Prades et al. ([2014](#_ENREF_14)).

| **Respondent ID** | **Raw responses*** | | **Ratio of Means (RoM)** | | **Median of ratios** | |
| --- | --- | --- | --- | --- | --- | --- |
|  | **X** | **Y** |  |  | **X based** | **Y based** |
| 1 | 25 | 100 | 1.00 | 0.25 | 0.25 | 4.00 |
| 2 | 10 | 100 | 1.00 | 0.10 | 0.10 | 10.00 |
| 3 | 5 | 100 | 1.00 | 0.05 | 0.05 | 20.00 |
| 4 | 100 | 5 | 0.05 | 1.00 | 20.00 | 0.05 |
| 5 | 100 | 10 | 0.10 | 1.00 | 10.00 | 0.10 |
|  |  |  | Mean=0.63 | Mean=0.48 |  |  |
|  |  |  | **RoM (X/Y)** | **1.31** | **0.25** | **4.00** |
|  |  |  | **RoM (Y/X)** | **0.76** |  |  |

# (*) Number (Ni) of patients X(Y) which are considered equivalent to 100 patients Y(X).
